# Laccase-catalyzed lignin depolymerization in deep eutectic solvents: challenges and prospects

**DOI:** 10.1186/s40643-023-00640-9

**Published:** 2023-03-23

**Authors:** Man Zhou, Olugbenga Abiola Fakayode, Manni Ren, Haoxin Li, Jiakang Liang, Abu ElGasim Ahmed Yagoub, Zhiliang Fan, Cunshan Zhou

**Affiliations:** 1 School of Food and Biological Engineering, Jiangsu University, Zhenjiang, 212013 People’s Republic of China; 2 Department of Agricultural and Food Engineering, University of Uyo, Uyo, 520001 Akwa Ibom State Nigeria; 3 Department of Food Science and Nutrition, King Saud University, Riyadh, 11451 Saudi Arabia; 4 Biological and Agricultural Engineering Department, University of California, Davis, 95616 USA

**Keywords:** Lignin depolymerization, Deep eutectic solvents, Laccase, Biocatalysis, Lignocellulosic biomass

## Abstract

Lignin has enormous potential as a renewable feedstock for depolymerizing to numerous high-value chemicals. However, lignin depolymerization is challenging owing to its recalcitrant, heterogenous, and limited water-soluble nature. From the standpoint of environmental friendliness and sustainability, enzymatic depolymerization of lignin is of great significance. Notably, laccases play an essential role in the enzymatic depolymerization of lignin and are considered the ultimate green catalysts. Deep eutectic solvent (DES), an efficient media in biocatalysis, are increasingly recognized as the newest and utmost green solvent that highly dissolves lignin. This review centers on a lignin depolymerization strategy by harnessing the good lignin fractionating capability of DES and the high substrate and product selectivity of laccase. Recent progress and insights into the laccase–DES interactions, protein engineering strategies for improving DES compatibility with laccase, and controlling the product selectivity of lignin degradation by laccase or in DES systems are extensively provided. Lastly, the challenges and prospects of the alliance between DES and laccase for lignin depolymerization are discussed. The collaboration of laccase and DES provides a great opportunity to develop an enzymatic route for lignin depolymerization.

**Graphical Abstract:**

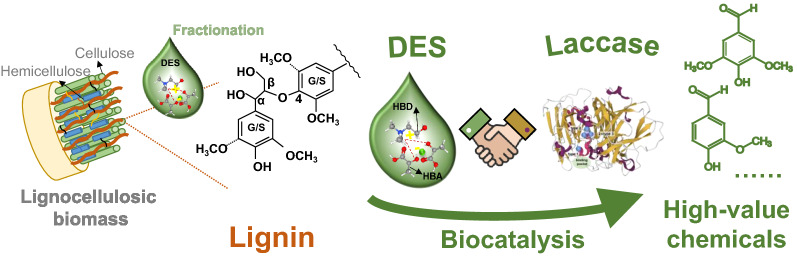

## Introduction

### Enzymatic depolymerization of lignin

Lignin is a heterogeneous and hydrophobic biopolymer composed of phenylpropanoid units through diverse C–C or C–O linkages. It accounts for almost 30% of the organic carbon on Earth (Curran et al. 2022; Rahimi et al. 2014; Yaguchi et al. 2021). Lignin can be efficiently fractionated from various agricultural wastes such as watermelon rind (Fakayode et al. 2020), sugarcane bagasse (Ji et al. 2021), corncob (Ma et al. 2022), and walnut shell (Li et al. 2023). Lignin is the most recalcitrant component among the three components of lignocellulosic biomass, namely cellulose, hemicellulose, and lignin. Currently, most majority of lignin is combusted to generate energy. However, it could be converted into various high-value products, including aromatic chemicals (such as vanillin) and other marketable products (Rahimi et al. 2014; Zevallos Torres et al. 2020). Depolymerization is considered one of the critical challenges in lignin valorization.

**Fig. 1:**
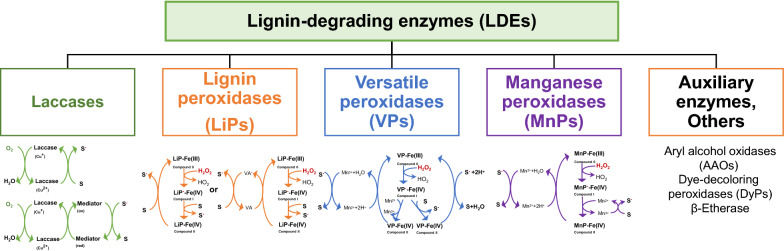
Classification of lignin-degrading enzymes and their schematic representations

Lignin depolymerization can be achieved through thermochemical, electrochemical, and biological methods (Zhou et al. 2022a). Generally, biological depolymerization of lignin is implemented under mild conditions and can enhance product selectivity by the inherent specificity of biocatalysts (Stevens and Shi 2022; Yaguchi et al. 2021). Numerous lignin-degrading enzymes (LDEs) have been discovered to break different linkages in lignin (Fig. 1) (Agrawal et al. 2018; Brugnari et al. 2021; Curran et al. 2022). The primary essential LDEs include laccase, lignin peroxidases (LiPs), versatile peroxidases (VPs), and manganese peroxidases (MnPs). Numerous auxiliary enzymes like aryl alcohol oxidases (AAOs) and other enzymes are involved in lignin depolymerization (Wang et al. 2020).

To develop biocatalytic processes, the reaction media must be optimized concurrently with the development of new enzymes (Itoh and Takagi 2021). An ideal solvent in the biocatalytic process must meet a series of criteria, such as good substrate solubility, good enzyme activity and stability, and favorable impacts on reaction equilibrium (Patzold et al. 2019). An ideal solvent can drive the reaction equilibrium to the expected direction and suppress the occurrence of side reactions. In biocatalytic processes, water is the most common solvent. However, the limited solubility of lignin in water impedes its bioconversion. Considerable efforts have been devoted to developing efficient solvent systems for lignin dissolution. Ionic liquids (ILs) and deep eutectic solvent (DES) are two solvent systems that have been successfully exploited and applied (Liu et al. 2019a). DES is sometimes called 4th generation ILs since they show better biodegradability and sustainability, more extensive availability, and are more affordable than ILs (Patzold et al. 2019; Sheldon 2021). Herein, DES is considered the preferred solvents for lignin depolymerization due to their low costs, green and sustainable advantages together with the high solubility of lignin (Zhou et al. 2023).

### Potential of laccase in lignin depolymerization

Laccases (benzenediol: oxygen oxidoreductases, EC 1.10.3.2) are multicopper oxidases that catalyze the four-proton reduction of O_2_ to H_2_O accompanied by the one-electron oxidation of four substrate molecules (Fig. 1). Two features make laccase more attractive than other LDEs: (1) the use of O_2_ instead of H_2_O_2_ result in a milder chemical environment, and (2) the production of water as the only by-product. Laccases are distributed throughout plants, fungi, bacteria, and insects. Laccase can directly oxidase the phenolic moieties of lignin. Furthermore, with the help of small-molecule mediators [e.g., 1-hydroxybenzotriazole (HBT), 4-hydroxybenzoic acid, 2,2′-azino-bis(3-ethylbenzothiazoline-6-sulfonic acid), ABTS], laccase enables the oxidation of lignin non-phenolic moieties (Canas & Camarero 2010). The small-molecule mediator can be paired with a laccase to develop a laccase mediator system (LMS). LMS can degrade 80 ~ 90% of the lignin structure (Chio et al. 2019; Munk et al. 2015). Because of its smaller size, once oxidized by laccase via electron abstraction, the small-molecule mediator diffuses away from laccase’s catalytic pocket and oxidizes the non-phenolic subunit of lignin. Furthermore, non-phenolic subunits can be catalyzed because oxidized mediators become highly reactive intermediates with redox potentials greater than non-phenolic lignin subunits. Generally speaking, mediators can help overcome the steric hindrance between the substrate and the enzymes as well as the high reduction potential of the substrate (Chan et al. 2019).

**Fig. 2:**
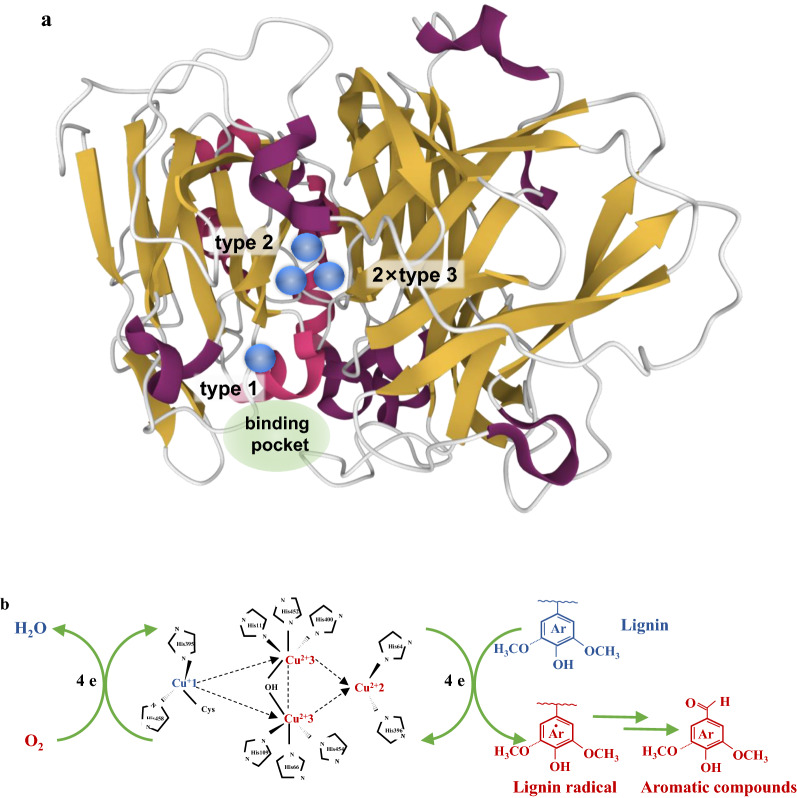
Structure of laccase from *Trametes versicolor* (PDBID: 1GYC) with the coppers (blue spheres) labeled by type and the substrate binding cleft highlighted in green (**a**), and lignin depolymerization catalyzed by laccase (**b**)

The moieties of lignin undergo one-electron oxidation by electron transfer to the blue type 1 copper, which is located near the substrate binding pocket (Fig. 2a). The unusual geometry around the type 1 copper is mainly responsible for its high potential. Two His and one Cys are arranged roughly trigonally around the copper, and two non-coordinating (or weakly) residues sit within about 0.4 nm in the axial positions. Type 1 copper is rapidly re-oxidized by long-range intramolecular electron transfer to the trinuclear copper cluster through a highly conserved His-Cys-His motif (Fig. 2b). Molecular oxygen binds between the two type 3 coppers and is reduced to water, which is coupled to the oxidation of the substrate (Valles et al. 2020). During the catalytic cycle, type 1 copper is oxidized and reduced four times (Rodgers et al. 2010).

**Fig. 3:**
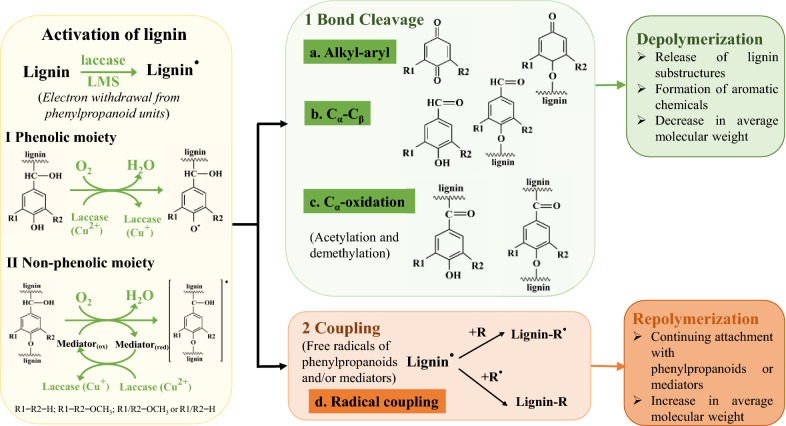
Possible changes of lignin during laccase catalyzation

The depolymerization of lignin by laccase or LMS is initiated via abstracting a single electron from the phenylpropanoid unit in lignin (Perna et al. 2019; Zhou et al. 2022b). This abstraction activates the lignin surface via forming an active radical, making lignin more reactive (Fig. 3). Since lignin is rich in conjugated bond systems, the unpaired electron in lignin radicals can delocalize to areas where the structure is most stable. The phenolic moieties of lignin are catalyzed to phenoxy radicals by laccases, and the non-phenolic moieties are oxidized to β-aryl radicals and benzylic radicals by LMS. These radicals are reactive intermediates and trigger two outcomes, as shown in Fig. 3. The first outcome is the consecutive cleavage of linkages (such as alkyl–aryl, C_α_–C_β_, and C_α_–C_1_) within the lignin polymer. These cleavage actions eventually lead to the release of lignin substructures, the formation of aromatic chemicals, and the reduction in average molecular weight (Munk et al. 2015). Some lignin degradation products in this process, for example, *p*-hydroxybenzoic acid and vanillin, act as mediators pairing with laccase and facilitate the further oxidation of the C_1_ carbon of β-O-4 and C_α_-C_β_ bonds in lignin (Yang et al. 2019). The second outcome is the coupling of lignin radicals and/or mediator radicals. Free radicals of phenylpropanoids and/or mediators are attached together via radical coupling reactions. This event will increase the average molecular weight of lignin and finally result in the repolymerization of lignin.

### Deep eutectic solvents as efficient solvents in biocatalysis

**Fig. 4:**
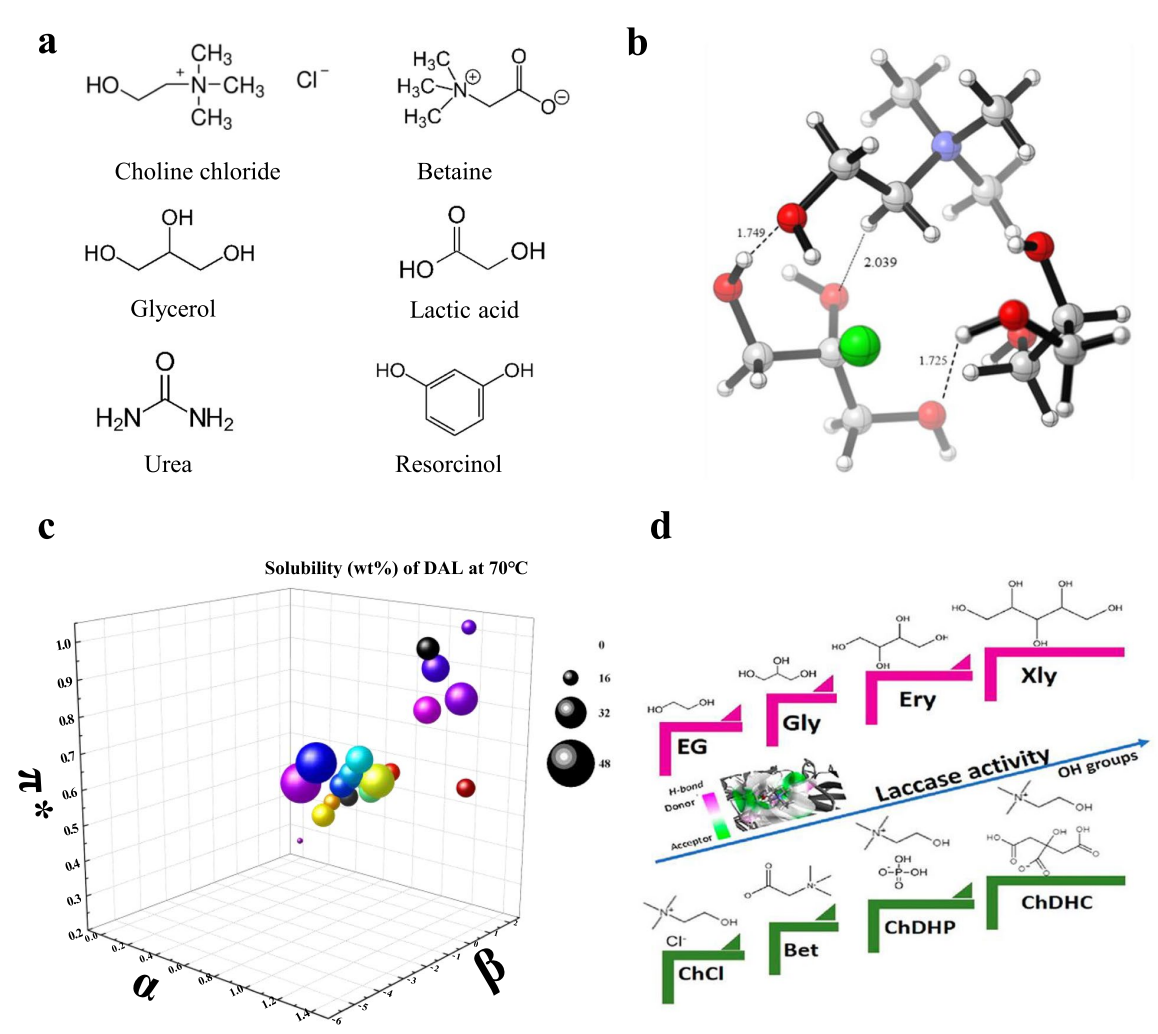
Representative structures of HBD and HBA (**a**), optimized geometry of ChCl:glycerol (**b**) (Nian et al. 2019), the correlation between lignin solubility and the K–T solvatochromic parameters of DES (**c**) (Zhou et al. 2022a, b), and laccase activity in different DES (**d**) (Toledo et al. 2019)

Deep eutectic solvents are eutectic mixtures that consist of hydrogen bond acceptors (HBAs) and hydrogen bond donors (HBDs) in a particular ratio (Zhou et al. 2023). They are commonly prepared by using natural precursors like choline chloride (ChCl) and betaine, and integrated with various HBDs like glycerol, lactic acid, urea, and resorcinol (Fig. 4a). DES shows a liquid state at ambient temperatures owing to the presence of intermolecular hydrogen bonds (H-bonds), lowering its freezing point. As shown in Fig. 4b, complex hydrogen bonding interactions between ChCl and glycerol had been formed. These interactions between O-H6 and O-H7, O-H8, and C-H2 play a vital role in the structural stability of ChCl–glycerol (Nian et al. 2019).

Because there are numerous possible combinations of HBDs and HBAs, a DES can be intelligently designed for a particularly enzymatic reaction. The behaviors of enzymes in DES depend on the nature of HBDs and HBAs (Patzold et al. 2019). Due to the strong H-bonds, DES tend to be very viscous, affecting mass transfer in catalytic transformations. Therefore, DES are often mixed with other solvents like water. The introduction of water into DES influences the interactions between enzymes and DES (Alizadeh et al. 2020; Rozas et al. 2021). For instance, through tailoring the hydrogen bonding interaction among DES via incorporating water, *Candida antarctica* lipase B was activated and stabilized (Nian et al. 2019).

The abundant presence of DES in all living cells convincingly demonstrates that DES works as the reaction media in synthesizing secondary metabolites, for example, flavonoids which are water-insoluble (Sheldon 2021). In brief, the biocatalytic processes in DES combine the advantages of the two worlds, namely the environmentally friendly of DES, and the outstanding selectivity and efficiency of enzymes (Zhang et al. 2021). Many biocatalytic processes have been developed using DES as solvents, co-solvents, or additives (Ghobadi & Divsalar 2020; Khlupova et al. 2021; Makkliang et al. 2021; Nian et al. 2019; Patzold et al. 2019; Peng et al. 2018). Generally, experimental and computational methods are adopted in concert to investigate the physicochemical parameters of biocatalysis processes, including viscosity, water activity (Bittner et al. 2022; Chan et al. 2021; Huang et al. 2020; Rozas et al. 2021), and oxygen transfer rate (OTR) (Zhang et al. 2021).

### Rationale for pairing laccase and DES for lignin depolymerization

Efficient lignin dissolution is a prerequisite for developing effective biocatalytic lignin depolymerization processes. In order to dissolve lignin in a solvent, it is typically necessary to break the π–π stacking interactions among lignin aromatic groups and create new H-bonds between the solvent and lignin. Molecular dynamics (MD) simulation analyses disclose that functional groups and oxygen atoms amount in the HBD of a DES determine its hydrogen bonding network as well as the strength of interactions with lignin (Li et al. 2021). Recently, several studies have indicated that lignin is highly dissolved in various DES, and their capacities of lignin dissolution substantially depend on their nature (Liu et al. 2019a; Malaeke et al. 2018; Soares et al. 2019). The lignin solubility in certain DES correlates closely with its K–T parameters (Fig. 4c). The lignin solubilities of type III DES showed a positive correlation with the subtraction value of α and β (Liu et al. 2019a). Given the ease with which the K–T parameters for DES can be tuned and measured, there is an enormous promise to develop suitable DES for the efficient dissolution of lignin.

Another prerequisite to developing the enzymatic conversion process is the tolerance of laccase toward the DES that efficiently dissolve lignin. Surprisingly, some laccases showed good biocompatibility with a wide range of DES (Fig. 4d). The activity of *Trametes versicolor* laccase was highly retained and even increased up to 200% in some DESs (Toledo et al. 2019). While laccase activity was partially lost in certain DESs (Khodaverdian et al. 2018), its biocompatibility with DES can be potentially promoted via enzyme engineering. Since the activity of laccase in ILs can be enhanced by protein engineering, its activity in DES can be improved through the same approach, given the similarity between DESs and ILs (Itoh and Takagi 2021; Sheldon 2021; Stevens and Shi 2019). Since laccase can depolymerize lignin into aromatic chemicals or other value-added products in aqueous ILs (Stevens et al. 2019), it is highly feasible for laccase to depolymerize lignin when utilizing DES as the reaction media.

Respecting the fact that DES highly solubilizes lignin and is biocompatible with laccase, the confluent utilization of DES and laccase has considerable promise for the establishment of new lignin depolymerization strategies. The combination of DES and laccase catalytic depolymerization further reinforces the sustainability of lignin biorefinery. Compared to the harsh thermochemical depolymerization of lignin, the biochemical process that uses laccase in DES is relatively gentle. It helps to channel desired aromatic compounds by facilitating the selective breakdown of lignin and maintaining the functionality of oligomeric lignols. Although laccase behaviors in DES have previously been investigated, there is still a fundamental gap in our knowledge of the processes driving the interactions between laccase and DES. It is necessary to conduct more research to determine how the solvent’s characteristics affect the lignin solubility and laccase activity in aqueous DES systems. Moreover, two areas require more research: (1) the exploration of DES-tolerant laccase, and (2) the development of biocompatible DES with increased lignin solubility. These advancements depend on thorough comprehending the interplay between laccase and DES, such as the structural modifications, structure–function relationship, dynamics, and OTR in DES.

### Scope of review

Recently, there have been several reviews on the effects of DES on enzymes (Patzold et al. 2019), the effects of ILS on LDEs for lignin valorization (Stevens and Shi 2019), and the effects of ILs on laccases (Itoh and Takagi 2021). To the best of our knowledge, yet no review about the effects of DES on laccases for lignin depolymerization has been published. This current review is focused on the fundamental interactions between DES and laccase, protein engineering strategies for improving DES compatibility with laccase, and controlling the product selectivity of lignin depolymerization by laccase in DES systems. The challenges and prospects of the association between DES and laccase for lignin depolymerization are critically discussed. Overall, this review aims to fill in some gaps in the study of implementing laccase in DES for the green and sustainable depolymerization of lignin.

## Interactions between laccase and DES

Profound knowledge of the interactions between laccase and DES is fundamental while aiming to develop a laccase–DES system that efficiently degrades lignin. So far, significant interests have gained attention for elucidating the interactions between laccase and DES through experimental or computational approaches or their combinations (Table 1). Many techniques from different aspects can study the interactions between laccase and DES: the activity of laccase in DES can be measured by colorimetric activity assays (CAA) using a wide variety of substrates such as ABTS and 2,2,6,6-tetramethyl-1-piperidinyloxy (TEMPO), the stability of enzymes can be experimentally determined by CAA and differential scanning calorimetry (DSC), the conformational changes and structural mobility can be obtained through spectroscopy usage of fluorescence, circular dichroism (CD), nuclear magnetic resonance (NMR), and small-angle neutron scattering (SANS) (Sanchez-Fernandez et al. 2021). Furthermore, computational techniques such as molecule docking simulations can provide complementary data and substantial molecular insights into the interactions between enzymes and DES.

**Table 1:** Recent advances in elucidating the interactions between laccase and DES

Laccase source	DES composition	Characterization methods	Main results	References
*Bacillus* HR03	ChCl-based and betaine-based NADESs aqueous buffer	CAA (substrate: ABTS); fluorescence spectrocopy	Enhanced 300%, 20% (v/v) betaine:glycerol	(Khodaverdian et al. 2018)
*Trametes versicolor*	Sixteen DES aqueous solutions (10, 25 and 50 wt%)	CAA (substrate: ABTS); molecular docking	1. ChCl-based DESs led to a decrease in the enzyme activity, while betaine-based DESs enhanced activity; 2. Laccase activity was dependent on the number of hydroxyl groups present in the polyols and their ability to H-bond with the enzyme amino acids; 3. The establishment of stronger H-bonds between DES components and laccase were responsible for the enhanced laccase activity	(Toledo et al. 2019)
	Nine aqueous NADES media	CAA (substrate: ABTS)	The thermostability of laccase improved in aqueous betaine-based NADES media (25 wt%)	(Delorme et al. 2020)
*Trametes hirsuta*	Three betaine-based DES (1:2) and aqueous media	CAA (substrate: ABTS)	1. Laccase completely lost its activity within 3 h in pure betaine-based DESs 2. In a 10% *v*/*v* betaine:glycerol (1:2) solution, laccase activity increased by up to 140%	(Khlupova et al. 2021)
*Myceliophthora thermophila*	Four DES aqueous media (ChCl:LA, ChCl:glycerol, betaine:glycerol, betaine:LA)	CAA (substrate: ABTS); kinetics; DSC; Crystallographic analysis; Fluorescence and CD spectroscopies	1. Laccase activity was enhanced up to 300% at a 2–8% *v*/*v* solution of betaine:LA; 2. ChCl:glycerol was a noncompetitive S-parabolic-I-parabolic mixed inhibitor of laccase; 3. DES tirggered changes in the local environments of the amino acids in the active site of laccase	(Chan et al. 2021)
*Pleurotus ostreatus*	Five betaine-based NADESs at a concentration of 25 wt% Sorbitol (Sor), xylitol (Xyl), glycerol (Gly), ethylene glycol (EtG), and erythritol (Ery)	CAA (substrate: ABTS); molecule docking	1. NADES is better than its individual components 2. The binding energies between laccases and NADES components correlate with the stabilization of laccases 3. The stabilization of interactions on enzyme surface, especially in flexible loops, was important to improve enzyme thermostability	(Varriale et al. 2022)

### Laccase activity and stability in DES

The activity and stability of laccase in DES depend on the chemical structure of HBA or HBD compounds, their molar ratio, and the exogenous addition of water (Toledo et al. 2019). These factors influence the properties of DES or DES–water aqueous solutions like viscosity, *a*_w_, OTR, volumetric mass transfer coefficient, and mass transfer coefficient (Zhang et al. 2021). Therefore, they affect the comprehensive interactions of enzymes with DES and, ultimately, the catalytic efficiency of the biocatalytic process. Compared with other enzymes, the laccase-catalyzed reaction may be more easily and strongly affected by the solvent medium. This is due to that the surface region of the laccase is in the proximity of its active site (Itoh and Takagi 2021; Varriale et al. 2022).

#### HBA or HBD

The functional groups and their amounts in DES influence laccase activity and stability. A variety of research reported that ChCl-based DES decreased laccase activity, while betaine-based DES increased it (Chan et al. 2021; Delorme et al. 2020; Khlupova et al. 2021; Khodaverdian et al. 2018; Toledo et al. 2019; Varriale et al. 2022). A detailed kinetic study showed that ChCl:glycerol DES was an S-parabolic-I-parabolic mixed noncompetitive inhibitor of laccase (Chan et al. 2021). The reduction of laccase activity in ChCl-based DES might originate from the chloride anion (Cl^−^). There are two possible explanations: (1) Cl^−^ is a chaotrope that destabilizes enzymes, and (2) Cl^−^ binds near the T1 Cu or surface of laccase and therefore inhibits its activity (Valles et al. 2020). Conversely, laccase in betaine-based DES was more stable and efficient than in aqueous solutions of their constituents (Delorme et al. 2020; Khodaverdian et al. 2018; Varriale et al. 2022). Although betaine could protect laccase via the strengthened H-bonds and electrostatic interactions (Mojtabavi et al. 2021), these findings illustrate a concerted effect of combing the HBA and HBD into a DES to play a thermostabilizing medium for laccase. Furthermore, the thermostability of laccase had been reported to be dependent on the H-bond interaction between the hydroxyl groups of HBD in the aqueous DES solutions and the amino acids in laccase (Delorme et al. 2020; Toledo et al. 2019). For example, Toledo and co-workers observed that both experimental results of relative activity and computation calculation of docking affinity energies for laccase with HBDs increased with the number of hydroxyl groups in the HBD (Toledo et al. 2019).

#### Molar ratio

The activity and stability of the laccase in DES also depend on the molar ratio of HBA and HBD in DES and their interactions with the laccase (Delorme et al. 2020; Khodaverdian et al. 2018; Toledo et al. 2019). The activity of laccase from *T. versicolor* in ChCl-based DESs decreased with the decreasing molar ratio of HBA: HBD (Toledo et al. 2019). This is because the concentration of Cl^−^ in ChCl-based DESs reduced with the decrease in the HBA: HBD molar ratio, thereby lowering the inhibition of Cl^−^ on laccase activity. Additionally, the molar ratio of betaine:xylitol had a marked impact on the thermostability of *T. versicolor* laccase (Delorme et al. 2020). Likewise, the activity of laccase from *Bacillus* HR03 in aqueous solutions of betaine:glycerol with three different molar ratios followed in the order—betaine:glycerol (1:2) > betaine:glycerol (1:1) > betaine:glycerol (1:3) (Khodaverdian et al. 2018).

#### External addition of water

**Fig. 5:**
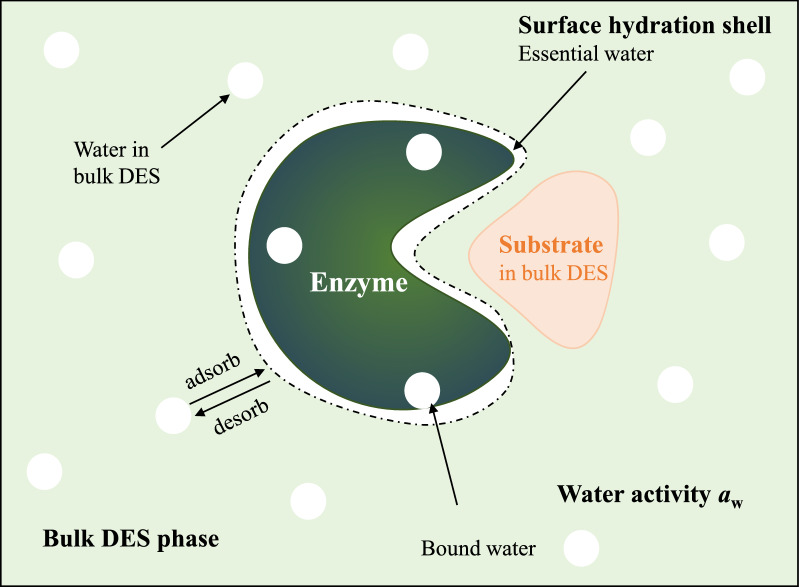
Illustration of the distribution of water around an enzyme (e.g., laccase) in a DES environment. Poor water-soluble substrate (like lignin) is efficiently dissolved in bulk DES. The space around the laccase can be distributed into two different regions: (1) an enzyme vicinity region, where the interaction of water with the laccase is dominant, and (2) a bulk DES phase region, where the interaction of water with the DES mixture dominates. Essential water layer might dwindle or disappear due to water stripping by hydrophilic DES

Water molecules affect an enzyme’s folding, structure, and function. Therefore, water molecules are essential for enzyme’s catalytic activity. Water molecules are spread between the enzyme surface, and the bulk DES phase in a system containing a laccase, water, and a DES (Fig. 5). Water molecules associated with the laccase can be defined as two types: (1) water buried inside the laccase is known as “bound water” (or “structural water”), which functions as a reactant and is an integral part of enzyme structure allowing stereospecific interactions; (2) water within the surface hydration shell of the laccase is referred to as “essential water” (or “free water”) because it enables laccase to maintain sufficient conformational flexibility for catalysis (Bittner et al. 2022; Zhao 2020). Water molecules incessantly ‘‘adsorb’’ onto and ‘‘desorb’’ from the laccase surface due to constant exchanges between the laccase and the bulk DES phase. The interaction of water with the bulk DES phase (i.e., *a*_w_) significantly influences the equilibrium of this constant exchange (Huang et al. 2020). Compared to the reference water, the *a*_w_ values of pure DES are relatively reduced (Zhang et al. 2021).

Increased water content has massive impacts on the laccase-catalyzed reactions in DES. First, water directly influences laccase activity and stability in DES. The *a*_w_ value has been shown to provide a reasonable estimate of oxidoreductase activity because it determines the enzyme water hydration layer and flexibility (Huang et al. 2020). Chan and co-workers revealed that more bound water molecules gathered in the laccase crystal structure in ChCl: LA environment (Chan et al. 2021). These laccase-bond water molecules were located on both the enzyme surface and interior, forming a hydration layer around the laccase. The additional water molecules are predicted to form intermolecular and intramolecular H-bonds with the laccase molecule, resulting in increased stability. Khlupova and co-workers found that laccase from *T. hirsuta* completely lost its activity within 3 h in pure betaine-based DES, while the activity increased to 140% in 10% *v*/*v* betaine:glycerol (1:2) solution (Khlupova et al. 2021).

Furthermore, the supramolecular network and physicochemical properties of DES are dependent on the water content (Gutiérrez et al. 2021; Nian et al. 2019; Rozas et al. 2021; Varriale et al. 2022). Generally speaking, the addition of water interrupts the original H-bond interaction of DES and the extent of interruption increases with water content. Meantime, new H-bonds among water and DES components form, altering the overall structure of DES–water. Hammond and co-workers characterized the nanostructure of a series of ChCl:urea:water DES mixtures by neutron total scattering and empirical potential structure refinement (Hammond et al. 2017). At low water content (ca. 42 wt% H_2_O), the DES nanostructure is retained because of solvophobic sequestration of water into nanostructured domains around [Ch]^+^. At 51 wt% H_2_O, the DES structure is disrupted. Instead, water–water, and DES–water interactions dominate. At and above this hydration level, the DES–water mixture is best described as an aqueous solution of DES components.

Similar results are found in ChCl:lactic acid (Gutiérrez et al. 2021) and ChCl: ethylene glycol (Rozas et al. 2021) by MD simulation. Water monomers are confined into the DES voids at low water content, interacting with the surrounding solvent via hydrogen bonding without significantly disrupting the solvent structural properties but increasing molecular mobility and decreasing H-bond lifetimes. Because the formation of water aggregates affects, albeit in a minor way, the DES structuring at higher concentrations, the disruptive effect of water molecules is greater (Gutiérrez et al. 2021). MD simulations revealed various H-bonds in these mixtures, formed between [Ch]^+^, Cl^−^, ethylene glycol molecules, and water (Alizadeh et al. 2020). The addition of water shifts the [Ch]^+^-Cl^−^ interaction toward more H-bonds. The alteration of supramolecular structures results in substantial changes in their physical properties. Recently, Varriale and co-worker have reported that the laccase activity enhanced in the betaine:sorbitol:water system (Varriale et al. 2022). Therefore, the amount of water in DES should be carefully tailored to maximize the laccase’s activity and stability sufficiently.

### Effect of DES on the structure of laccase

The biological activity and stability of laccase are closely related to its structural conformations. Although a wide range of DESs have recently been investigated for their ability to retain the activity and stability of laccases, only several studies have centered on the structural changes of laccase in these solvents (Chan et al. 2021; Khodaverdian et al. 2018). Khodaverdian and co-workers reported that the fluorescence spectra of *Bacillus* HR03 laccase in different DESs were quite different in terms of maximum fluorescence intensity (*I*_max_), fluorescence intensity, and shift of maximum fluorescence wavelength (*λ*_max_) (Khodaverdian et al. 2018). Different DESs exhibited diverse effects on laccase conformations. For instance, laccase in betaine:glycerol showed similar *I*_max_ with buffer, whereas a reduction in *I*_max_ was noticed in betaine:malic acid:water. Recently, Chan and co-workers found red-shifted emission spectra (increased *λ*_max_) of *Myceliophthora thermophila* laccase upon adding DES, thereby suggesting that DES induced a more flexible structure of laccase (Chan et al. 2021). Furthermore, the random coil content of *M. thermophila* laccase in ChCl:glycerol environment slightly rose by CD spectroscopies. Analysis of laccase crystal structure revealed that its trinuclear active site changed when adding ChCl:LA. However, the increased bond water molecules protected laccase from undergoing massive structural changes. In summary, the presence of DES substantially influences the structural conformations of laccase, and their structural changes depend on the corresponding nature of DES.

Molecular docking, a computational method, has been applied to gain a deeper insight into the mechanism of DES on laccase activity. Different types of molecular interactions, including H-bonds, electrostatic interactions, hydrophobic, cation–π, and ionic interactions, have been found in the optimum binding models for laccase–DES complexes (Toledo et al. 2019; Varriale et al. 2022). Toledo and co-workers performed molecular docking calculations to explore the optimal binding poses, docking affinity energies, interacting amino acids, and interaction types (Toledo et al. 2019). They revealed that both HBAs and HBDs formed hydrogen bonding with specific amino acids, namely serine, alanine, and histidine. Additionally, docking affinity energies between HBAs and laccase agreed well with relative laccase activities. The boosted laccase activity mainly stemmed from the interactions formed with the histidine amino acids at its catalytic cluster. Analogously, by calculating the binding energy spread of the optimum laccase–HBD couple conformation, Varriale and co-workers found that the residual activity of laccase in DES was in line with the binding energy when exposed to high temperatures (Varriale et al. 2022). The exposed region and inner portion of the L1 loop represent common targets for interactions with DES. Altogether, these findings suggest that molecular docking is a powerful approach to elucidate the interactions between laccase at the molecular level and DES. This approach can assist the tailoring of DES for laccase-catalyzed lignin deconstruction.

### Specific ion effect on laccase

**Fig. 6:**
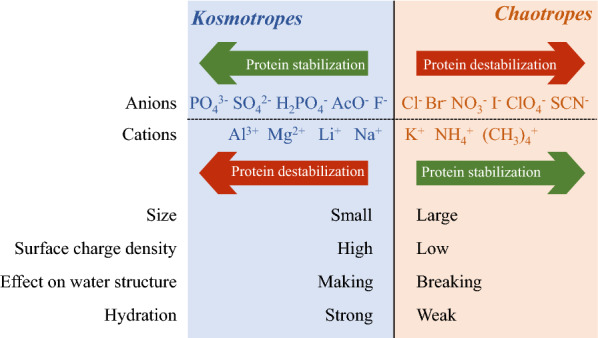
The Hofmeister series and classification of kosmotropic and chaotropic ions

The presence of charged ions in some DESs (like ChCl-based DESs) causes favorable solvation environments with peculiar ion–ion interactions between the ions and charged residues, thereby changing the dynamics and structure of enzymes (Sanchez-Fernandez and Jackson 2021). The interactions of enzymes with cations/anions in DES follow the Hofmeister or lyotropic series (Fig. 6) (Itoh and Takagi 2021; Sanchez-Fernandez et al. 2021). Kosmotropic anions are small-sized, highly charged, and strongly hydrated ions and commonly promote enzyme stabilization. In contrast, hydrophobic chaotropic anions, which are large-sized, low-charged, and weakly hydrated, result in enzyme destabilization (Sheldon 2021). Meanwhile, chaotropic cations can enhance the stabilization of enzymes because more chaotropic anions disrupt the water shell around an enzyme, resulting in enzyme destabilization.

For instance, the choline cation (Ch^+^) in ChCl (most commonly used HBA in DES) is expected to play an instructive role in increasing enzyme activity. In contrast, chloride anion (Cl^−^) has a destructive effect on the enzyme. When sugars (such as fructose, glucose, and sucrose) act as HBD, they behave as chaotropes at low concentrations and kosmotropes at high concentrations (Ghobadi & Divsalar 2020). Specific ion effects have been observed when using ChCl, choline dihydrogen phosphate, and choline dihydrogen phosphate as the HBA in DES solutions (> 50 wt% water) (Delorme et al. 2020; Toledo et al. 2019). Effects of anions on the activity and stability of laccase complied with the Hofmeister order (C_6_H_8_O_7_^−^ > H_2_PO_4_^−^ > Cl^−^) when paired with the same cation (Ch^+^). These findings indicate the feasibility of designing desirable DES for lignin bioconversion according to the Hofmeister or lyotropic order of respective cations and anions in DES (Sanchez-Fernandez et al. 2021).

## Protein engineering to improve the compatibility of DES with laccase

Numerous studies have reported that ChCl-based DESs that highly dissolve lignin hinder laccase activity (Chan et al. 2021; Delorme et al. 2020; Khlupova et al. 2021; Khodaverdian et al. 2018; Toledo et al. 2019; Varriale et al. 2022). A promising prospect for boosting the activity and resistance of laccases in DES is protein engineering (Wallraf et al. 2018). Table 2 summarizes the recent protein engineering efforts to improve laccase tolerance to ILs or other enzymes to DES.

**Table 2:** Summary of protein engineering strategies for improving tolerance of laccase to ILs or other enzymes to DES

Enzyme source	Approach	Characterization techniques	Main outcomes	Reference
*Trametes versicolor* laccase (Lcc2)	Directed evolution	CAA	4.5-fold higher activity in 15% (v/v) of [EMIM] [EtSO4] (M3 mutant)	(Liu et al. 2013)
	KnowVolution	CAA, computational modeling and evolutionary conservation analysis	Improved activity in ILs; Loop 1 was important for improving laccase resistance with ILs	(Wallraf et al. 2018)
*Pleurotus ostreatus* laccase	Directed evolution	CAA, and molecular modeling	Higher thermostability of laccase in acidic and alkaline pH and aqueous betaine-based NADESs	(Piscitelli et al. 2011; Varriale et al. 2022)
*Melanocarpus albomyces* laccase	KnowVolution	CAA with various substrates: ABTS, 2,6-dimethylphenol and syringaldazine; molecular docking and simulations	Improved stability and activity at alkaline pH; Key residues that located in close proximity of the T1Cu site were identified to increase alkaline tolerance	(Novoa et al. 2019)
*Myceliophthora thermophila* laccase	Combination of computational-assisted rational design and site-directed mutagenesis	MD simulations	The inhibition mechanism of [C_2_C_1_Im][OAc] toward *M. thermophila* laccase is likely not dependent upon the IL interacting with the enzyme surface	(Stevens & Shi 2022)
*Clostridium cellulovorans* cellulase	Directed evolution	CAA and MD simulations	Improved tolerance to ILs and DESs; Residue Arg300 was the key for the ionic strength activation through a salt bridge with the neighboring Asp287	(Lehmann et al. 2014)
*Penicillium verruculosum* cellobiohydrolase I	Combination of computational-assisted rational design and site-directed mutagenesis	CAA and MD simulations	Improved tolerance to DES; the formation of salt bridges and *π*–*π* interaction in variants stabilized surface exposed flexible α‐helix and highly flexible loop in the multi‐domain β‐jelly roll fold structure	(Pramanik et al. 2021)

### Rational design

**Fig. 7:**
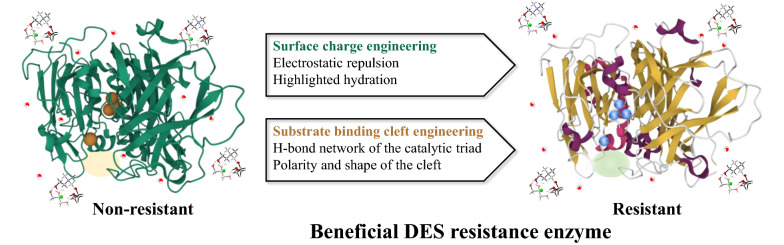
Rational design strategies to improve laccase resistance with DES

Rational design is considered universal and faster to deliver tailor-made enzymes. It requires knowledge of the residues in charge of improving an enzyme's tolerance to nonaqueous media (like ILs and DESs) (Stevens and Shi 2019). These residues are commonly identified using a homolog model by computational analysis. The surface loop 1 and the substrate bind cleft of laccase have been reported as the interaction regions between laccase and DES (Chan et al. 2021; Toledo et al. 2019; Varriale et al. 2022). Key residues governing ionic strength resistance were identified, and a molecular mechanism for salt activation via a salt bridge was proposed and validated. Interactions between DES components and the side chain amino acids of substrate binding cleft caused laccase to rearrange its conformation, making its catalytic domain more approachable to substrates (Toledo et al. 2019). Introducing acidic amino acid at loop 1 can increase DES resistance through ionic interactions with salt ions. Based on these findings, two rational design strategies, surface charge engineering and substrate binding cleft engineering (Fig. 7), have been proposed to increase laccase resistance in DES.

Recently, a few reports have demonstrated that surface charge engineering and substrate binding cleft engineering efficiently enhance enzyme activity in ILs (Pramanik et al. 2022; Stevens and Shi 2019; Wallraf et al. 2018). The enhanced enzyme hydration shell and electrostatic repulsion of ions are the two primary drivers to enhance ILs resistance (Pramanik et al. 2022). For instance, based on the computational assisted surface loop engineering, two variants—OM1 and OM3 experienced a 3.9-fold and 1.6-fold improved specific activity than wild-type in aqueous solutions of ILs, respectively (Wallraf et al. 2018). Similarly, the thermostability of laccase from basidiomycete PM1 was doubled by modifying the flexible surface loops in laccase (Vicente et al. 2019). Additionally, since that DESs are the 4th of ILs, further possibilities of improving the compatibility of laccase in DES through flexible surface loops charging engineering and substrate binding cleft can be envisioned.

### Directed evolution

**Fig. 8:**
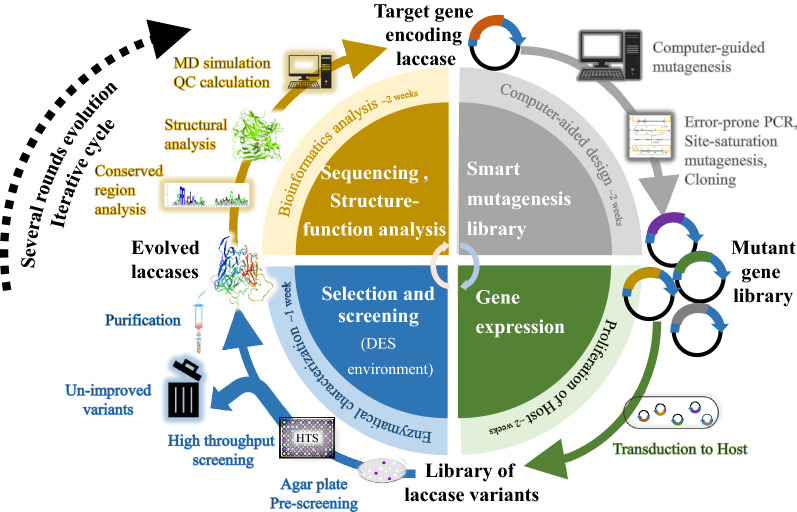
Schematic representation of the directed evolution workflow of improving the laccase resistance with DES

Directed evolution is a powerful strategy to develop enzymes with improved characteristics or new functions. Figure 8 schematically shows the directed evolution workflow of improving the laccase resistance in DES. The pivotal techniques in directed evolution are the creation of genetic diversity through various mutagenesis methods (e.g., error-prone PCR, multi-site saturation mutagenesis, and computer-guided mutagenesis) and robust high-throughput screening (HTS) methods. Besides, the selective pressure is controlled artificially, and the evolutionary time scale is compressed to only a few weeks in the laboratory. For instance, while attempting to improve the resistance of laccase in DES, the selective pressure is the gradually increased concentration of DES aqueous solution with the iterative rounds of evolution (Fig. 8).

In order to balance screening efforts, improvements of enzyme properties, and a molecular understanding of improved properties, several combined strategies such as CAST/ISM, FRESCO, ProSAR, MORPHING, OmniChange, and KnowVolution have been developed (Bornscheuer et al. 2019; Cheng et al. 2015). These methods create “small but smart” mutant libraries coupled with advanced HTS methods on the bases of flow cytometry and microfluidic devices, and consequently balance throughput and time (Bornscheuer et al. 2019). By integrating computer-guided mutagenesis and directed evolution in conjunction with reliable HTS methods, a high-redox-potential laccase with improved redox potential and stability has been obtained (Mateljak et al. 2019). KnowVolution, a knowledge-gaining directed evolution strategy that allows the customization of enzyme characteristics with minimum experimental efforts by coupling directed evolution with computational analysis, has successfully been employed to improve laccase tolerance in ILs (Wallraf et al. 2018) and alkaline pH (Novoa et al. 2019).

Directed evolution can improve enzyme performance in the lack of enzyme structural or mechanistic information (Mate and Alcalde 2015). It has proven to be an effective method for discovering different aspects of stabilizing laccases in ILs (Liu et al. 2013; Wallraf et al. 2018) or other enzymes in DES (Lehmann et al. 2014; Pramanik et al. 2021). Two rounds of directed laccase evolution yielded an Lcc2 variant M3 which showed 4.5-fold higher activity than the wild-type in 15% (*v*/*v*) [EMIM] [EtSO_4_] ionic liquid solution (Liu et al. 2013). Lehmann et al. generated a ChCl:glycerol tolerant 1,4-endoglucanase mutant by combining directed evolution with computer-guided analysis (Lehmann et al. 2014). Although the molecular knowledge regarding how substitutions improve laccase activity in a DES has yet to be understood, directed evolution (e.g., KnowVolution) holds great potential to gain general and transferable design principles for improving laccase tolerance to DES.

## Selective depolymerization of lignin by laccase or in DES

Selective production of value-added products from the enzymatic conversion of lignin is of strategic importance (Shen et al. 2020). Due to the complex structure and high polydispersity of lignin, selective product formation from lignin depolymerization remains challenging (Stevens & Shi 2019). In recent years, a few scientific studies which focused on the tailorable depolymerization of lignin through laccase/LMS or in DES have been carried out (Table 3). These studies demonstrate that laccase and DES hold enormous potential in lignin depolymerization. Still, those existing studies have some limitations or drawbacks, as shown in Table 3. The main problems met by using the laccase or DES are as follows: (1) the low activity of laccase in some ILs or DESs (Delorme et al. 2020; Liu et al. 2019b; Toledo et al. 2019; Varriale et al. 2022), (2) the requirement of mediators for efficient lignin depolymerization by laccase (Zhu et al. 2020), (3) in some case, the combination of mediators with laccase reduces the selectivity for S-lignin over G-lignin (Vuong et al. 2021), different mediators maybe result in different selectivity (Kontro et al. 2020), and (4) some side effects such as repolymerization occur (Yu et al. 2020). The selective lignin degradation is rooted in the specificity of laccase/LMS and the tailorable characteristics of ILs/DESs. Additionally, undesired side reactions can be suppressed through various strategies to improve the overall yield of lignin depolymerization.

**Table 3:** Overview of scientific studies concerning laccase/LMS or DES on the selective depolymerization of lignin

Conditions of lignin depolymerization	Lignin type or source	Laccase origin or DES	Main results or limitations	References
Two-step chemoenzymatic depolymerization containing LMS/IL/Buffer and aqueous alkaline solution	Lignin from beech wood	*Trametes versicolor* (Lcc2-M3)	A *T. versicolor* laccase variant (Lcc2-M3) catalyzed the essentially selective α-oxidation of the β-O-4 linkage to β-hydroxyketones at room temperature	(Liu et al. 2019b)
Laccase-catalyzed degradation in ILs aqueous solution	Alkaline lignin	*Trametes versicolor* (Lcc2)	Alkaline lignin was majorly depolymerized into vanillin, acetosyringone, syringaldehyde, and acetovanillone	(Stevens et al. 2019)
Laccase-catalyzed degradation	Rice straw, corn stover, reed, kraft lignin, and organosolv lignin from rice straw	*Caldalkalibacillus thermarum*	*C. thermarum* laccase efficiently depolymerized lignin into seven high-value benzaldehyde chemicals from lignocellulosic biomass and commercial lignin samples	(Yang et al. 2019)
	Alkaline lignin and milled wood lignin	*Bacillus ligniniphilus*	The *B. ligniniphilus* laccase can effectively degrade G-lignin even without a mediator, and the removal rate of G-lignin is higher than that of S-lignin. With the aid of mediator, laccase inreceased the removal rate of H-lignin	(Zhu et al. 2020)
	Milled wood lignin	*Amycolatopsis* sp. 75iv2	The laccase degradade 58% of S-lignin over 16 h. The combination of ABTS with laccase reduced the selectivity for S-lignin over G-lignin	(Vuong et al. 2021)
Laccase-catalyzed degradation at low pH	Lignin model compounds and technical lignin	*Obba rivulosa*	In the presence of both N–OH-type and phenolic mediators, the laccases selectively oxidized lignin in acidic reaction conditions, and in the laccase-TEMPO system, the syringyl-type lignin units were preferred	(Kontro et al. 2020)
Electrochemical degradation of lignin in a DES system	Kraft lignin	ChCl:ethylene glycol and ChCl:urea	Guaiacol and vanillin were the two most abundant detected products	(Di Marino et al. 2016)
Metal-based DES catalysis of lignin	Organosolv lignin from herbaceous biomass	ChCl:FeCl_3_ (1:2)	Lignin was directedly degraded into methyl *p*-hydroxycinnamate as the sole product with high yield and selectivity (105.8 mg g^−1^ and 74.1%, respectively)	(Li et al. 2020)
Chemocatalysis of lignin in a DES system	Alkaline lignin	ChCl:methanol	A high total yield of acetovanillone and acetic acid (87.12%) was obtained from alkaline lignin under mild conditions	(Yu et al. 2020)
Catalytic hydrogenolysis using DES	Lignin from castor seed coats	ChCl:ethylene glycol, ChCl:glycerol, and ChCl:propylene glycol	High selectivity towards 4-propyl catechol was observed	(Liu et al. 2021b)
A combination of chemical depolymerization by DES and bioconversion by *Bacillus australimaris*	Alkali lignin	ChCl:glycerin	ChCl:glycerin released more soluble small molecules from lignin, and confirmed improvement in lignin valorization via the combination of chemical and biological methods	(Yu et al. 2021)
Catalytic hydrogenolysis and acidolysis using DES	Lignin from birch	ChCl:oxalic acid:ethylene glycol	The obtained ethylene glycol protected lignin displays high β-O-4 content and can be readily depolymerized to distinct monophenolic products	(Liu et al. 2021c)

### Directing product formation via laccase

Selective transformation of lignin into a pure single product or multiple products using various catalysts in DES has already been explored, including electrochemical (Cruz et al. 2022; Di Marino et al. 2017, 2016) or microbial (Liu et al. 2018; Zhu et al. 2020) catalysis. The use of enzymes could potentially offer improved selectivity and suppress undesired side reactions due to their stereoselectivity and regioselectivity (Chio et al. 2019). Laccases and LMS can selectively oxidize the subunits of lignin to reactive radical intermediates, leading to the cleavage of lignin polymer (Zhou et al. 2022b).

The redox potential *E°′* of laccase type 1 copper can modulate the selectivity of lignin degradation. Divergences in axial ligand coordination, electrostatic interactions, hydrophobicity, and total solvent accessible surface area are the reasons for the broad *E°′* of type 1 copper center (Vilbert et al. 2021). Three laccases from different origins showed different affinities and kinetic parameters for the same lignin substrate (Perna et al. 2019). Higher quantities of *p*-hydroxybenzaldehyde and vanillin were released from lignin degraded by *Caldalkalibacillus thermarum* laccase than *T. versicolor* laccase (Yang et al. 2019). Moreover, laccases show different preferences for different lignin subunits. For example, a bacterial laccase from *Bacillus ligniniphilus* preferentially degraded guaiacyl-lignin over syringyl-lignin (Zhu et al. 2020). Additionally, LMS can be used to tune the laccase for lignin degradation into different products. LMS increases the oxidative potential of laccase, thereby increasing the number of products obtained by expanding laccase to oxidize non-phenolic lignin structures (Chio et al. 2019). For instance, with the aid of both N–OH-type and phenolic mediators, the laccase from *Obba rivulosa* selectively oxidized lignin in acidic reaction conditions (Kontro et al. 2020). The syringyl-type lignin moiety was preferentially decomposed in an iron (III)–TEMPO mediated laccase oxidation system. However, the conjunction of ABTS with small laccase from *Amycolatopsis* sp. 75iv2 selectivity reduced the syringyl-lignin over guaiacyl-lignin (Vuong et al. 2021).

Although mediators can finely tune the yield and distribution of products from lignin depolymerization, the requirement of mediators may be a limitation in some cases. The first reason is the cost of exogenous mediators. Although the required amounts of redox mediators are catalytic, the cost cannot be overlooked (Zhou et al. 2022b). Another reason is that some mediators have inhibitory potential on laccase activity due to undesired side effects (Mate and Alcalde 2017). Side products formed after laccase oxidation deplete mediators from the LMS and obstruct the redox cycle between laccase and substrate. Furthermore, the covalent binding of mediators to the substrate can eliminate them from the reaction system. Besides, some synthetic mediators are potentially toxic. Strikingly, some aromatic chemicals derived from lignin depolymerization can serve as redox mediators (Canas and Camarero 2010). In this regard, implementing LMS based on natural mediators derived from lignin depolymerization is an area that deserves further investigation.

### Selective tailoring of lignin via DES

The product formation of lignin depolymerization is affected by its intrinsic properties, whereas the properties of lignin are significantly influenced by the DES used (Ji et al. 2021; Zhou et al. 2022a, 2023). To date, many efforts have been made to develop DES systems with higher selectivity to produce value-added chemicals from lignin (Table 3). Through electrochemically depolymerizing lignin in a DES system, guaiacol and vanillin were the two most abundant detected products (Di Marino et al. 2016). A metal-based DES was carefully designed and successfully applied to solely produce methyl *p*-hydroxycinnamate with great specificity (Li et al. 2020). The selectivity of catalytic oxidation of lignin into acetovanillone and acetic acid in a ChCl:methanol-based DES process was enhanced by raising the HBA/HBD molar ratio, which might be due to the high solubility of lignin in DES (Yu et al. 2020). A highly specific production of 4-propyl catechol was achieved in direct hydrogenolysis of C-lignin in DES using metal catalysts (Liu et al. 2021b). By simply tuning the relative ratios of the DES components, the reactivity of the β-O-4 moiety can be directed to either scission or protection (Liu et al. 2021c). These lignins delivered 6 times more aromatic monomers upon depolymerization than the condensed analogs obtained using binary (ChCl/OA) DES. Furthermore, the conductor-like screening model for real solvents (COSMO-RS), a computational method that significantly alleviates the experimental burden of selecting optimal solvents from a vast pool of DESs by computing partition coefficients and selectivity factors of lignin or its monomers, holds great potential in the designing of DES systems that depolymerize lignin with high product selectivity (Shen & Van Lehn 2020).

It has been demonstrated that the solvation of lignin in DES affects lignin reactivity (Li et al. 2021; Qiao et al. 2021). The functional groups alongside with oxygen atom quantities in the HBD of a DES jointly influence the features of DES’s hydrogen bonding network and interaction strength. DES dissolves lignin by altering H-bond network density and breaking the H-bonds in lignin (Li et al. 2021). The affinity and nucleophilicity of the β-O-4 linkage in lignin are affected by DES in three ways: (1) the formation of a charged solvation layer, (2) adjusting the exposure of the β-O-4 linkage allowing more catalysts to approach and depolymerize lignin, and (3) changing the electrostatic potential of the β-O-4 linkage (Qiao et al. 2021). Nonetheless, such a comprehensive relationship would present challenges in designing a suitable DES. Meanwhile, this multiscale relationship would also offer possibilities for designing DES with high selectivity to degrade lignin. Nevertheless, the depolymerization of lignin by laccase in DES media has been scarcely explored. The attempt involving lignin being successfully depolymerized by laccase in ILs into aromatic chemicals with high selectivity (Stevens et al. 2019) shed light on paring the product selectivity offered by both laccase and DES. DES holds massive potential in developing biocatalytic lignin conversion strategies with high selectivity.

### Lignin repolymerization prevention and product yield improvement

While dealing with lignin depolymerization, a primary issue is the repolymerization or condensation of lignin. For example, the depolymerization and condensation reactions of the side chain groups of alkaline lignin coincided in a ChCl:methanol-based DES system (Yu et al. 2020). As shown in Fig. 3, the active intermediates formed during lignin depolymerization, such as free radicals of phenylpropanoids and mediators, are susceptible to repolymerization and increase average molecular weight. Plant-derived laccases contribute to lignin polymerization processes. The crux of lignin valorization is to drive the direction of the overall reaction to depolymerization. Laccase with higher *E°′* favors depolymerization, whereas laccase with lower *E°′* encourages repolymerization (Chan et al. 2019). The *E°′* of laccase can be adjusted by protein engineering strategies or with the aid of mediators, which can be used to reduce or prevent depolymerization or condensation of lignin.

Preventing depolymerization products from repolymerization or condensation may improve the yields of products from lignin depolymerization. The most promising approach appears to be utilizing a capping agent as the active stabilizer to protect α, γ-diol in lignin. Formaldehyde can prevent lignin condensation by forming 1,2-dioxane structures with lignin side-chain hydroxyl groups, facilitating lignin monomer production (Shuai et al. 2016). Acetaldehyde and propionaldehyde have been shown to stabilize the α, γ-diol group, thus preventing lignin’s condensation and increasing the selectivity of products (Lan et al. 2018). Lignin condensation easily occurs in acidic conditions through dehydration of the α-OH followed by condensation of the resulting unsaturation with a neighboring aromatic group. When the α-OH is oxidized, it is no longer able to dehydrate, limiting lignin condensation. The oxidant/catalyst 2,3-dichloro-5,6-dicyano-1,4-benzoquinone was used to selectively deprotect the acetal and oxidize the α-OH into acetone (Lan et al. 2019). The deprotected β-O-4 units generated in situ were rapidly converted to α-ketones, which limited condensation and other structural modifications resulting in higher yield and product selectivity.

Another promising approach is the stabilization of reactive intermediates by the tunable and functional DES. Recently, Liu and co-workers developed ternary DES systems composed of ChCl, oxalic acid, and diols that prevented undesired lignin condensation (Liu et al. 2021c). The in situ trapping of the reactive C2-aldehydes originating from acidolysis of β-O-4 linkages by various diols resulted in cyclic C2-acetals and suppression of recondensation processes. By modulating DES composition, the reactivity of the β-O-4 linkage can be steered towards either cleavage or stabilization. Therefore, using a capping agent in conjunction with a laccase–DES pair could increase product yields while allowing for specific product formation.

The laccase amount and time required for the enzymatic depolymerization of lignin must also be investigated as potential roadblocks toward boosting yield. Most studies reported reaction times ranging from several hours to days for enzymatic degradation of lignin in water or ILs (Liu et al. 2019b; Stevens et al. 2019; Yang et al. 2019). Likely, the laccase quantities and reaction times required for enzymatic lignin depolymerization in DES will be comparable. Several strategies, including enzyme engineering for desirable laccases, process intensification and optimization for improved laccase stability and recyclability, and solvent engineering of DES for superior laccase performance and product selectivity, are promising to improve the efficacy of lignin depolymerization by laccase in DES media.

## Challenges and prospects

Although significant progress has been recently achieved in pairing laccase and DES for lignin depolymerization, a long road lies ahead, and the following research fields remain to be further explored:

1. It is critical to apply a variety of experimental methods and computational tools to investigate the laccase–DES interaction and how it affects laccase ligninolytic capabilities. Computational methods are usually adopted to gain a deeper understanding of interactions in the systems of laccase–DES, DES–lignin, or laccase–DES–lignin at the molecular level. For instance, in the systems of ChCl-based DES–laccase and ChCl-based DES–lignin, molecular docking revealed that H-bonds, electrostatic interactions, hydrophobic, cation–π, and ionic interactions play important roles in laccase function and lignin structure (Qiao et al. 2021; Toledo et al. 2019; Varriale et al. 2022). However, computational techniques present restrictions, such as the requirement of accurate force fields for MD simulations to refrain from mechanistic aberrations (Sanchez-Fernandez and Jackson 2021). Part of its solution is to use experimental results as constraints to refine simulation results. Therefore, the concise conclusion regarding the apparent inhibition of ChCl-based DESs on laccase activity should be drawn comprehensively based on both experimental and computational results: (i) The OTR of dioxygen from gaseous to ChCl-based DESs significantly decreased due to their high viscosities (Zhang et al. 2021); (ii) The inhibition of Cl^−^ on laccase is pH dependent, and the determination of pH optima for solution-based assays are problematic (Rodgers et al. 2010; Valles et al. 2020); (iii) The computationally calculated binding affinity energies between ChCl and laccase were much lower than those of other tested DESs (Toledo et al. 2019); (iv) MD simulations revealed that ChCl-based DESs formed a positive solvation shell around lignin that created a thermodynamic barrier for laccase binding (Qiao et al. 2021). A thorough understanding of laccase–DES–lignin interactions will aid in the fundamental knowledge of laccase stabilization in DES, the selectivity of lignin depolymerization in DES, and ultimately the design of laccases with improved DES resistance for lignin valorization.
2. Protein engineering strategies could be employed to enhance existing or create novel laccases that can withstand high concentrations of DES. The biocompatibility of DES is a significant factor that impacts their feasibilities in the enzymatic lignin depolymerization process (Liu et al. 2021a). The low activity/resistance of available laccase in the presence of ChCl-based DESs that highly dissolve lignin hinders the enzymatic conversion of lignin (Chan et al. 2021; Delorme et al. 2020; Khodaverdian et al. 2018; Toledo et al. 2019). However, laboratory evolution approaches for improving laccase resistance in DES systems have yet to be reported. In the future, considerable efforts should be taken to fill this research gap. The recent successful cases that enhance laccase tolerance in ILs or other enzymes in DES using various advanced protein engineering regimes (Table 2) have brought about a beam of new light to modify existing laccases with desirable resistance to DES. Furthermore, benefiting from the advancement of computational techniques and high-throughput sequencing, the targeted discovery of novel laccase through genome-wide identification and de novo design becomes achievable (Zhou et al. 2017). Although these milestones could be reached, utilizing laccase for scalable lignin valorization is still hampered by the high cost of mediators and enzyme production (Brugnari et al. 2021; Curran et al. 2022). New technologies, such as MPEPE (Ding et al. 2022), which highly enhance laccase heterologous expression, must be constantly developed toward laccase industrialization.
3. Testing the efficacy of lignin–DES–laccase systems on industrially relevant polymeric lignin or natural lignin feedstocks is vital. The substrate for laccase activity measurement is usually ABTS or simple lignin model compounds like 2,6-dimethylphenol. Their structures are much less complicated than lignin. Both actual laccase activity toward lignin and product distribution significantly differ from those of the model compounds. The catalytic performance of laccase on the actual lignin polymer substrate should be systematically and suitably evaluated. Solution-based spectrophotometric assays are unsuitable for measuring the oxidative rates of non-soluble lignin by laccase (Valles et al. 2020). Recently, advanced techniques such as 2D NMR, nanostructure-initiator mass spectrometry (NIMS), electron paramagnetic resonance (EPR) spectroscopy, and isothermal titration calorimetric (ITC) have been developed to study lignin depolymerization by laccase (Deng et al. 2018; Perna et al. 2019; ST et al. 2022). These methods present distinct advantages over solution-based spectrophotometric assays: NIMS offers abundant information on bond cleavage and quantitative analysis of product generation along with kinetics, EPR enables monitoring the kinetics of radical formation during the laccase-catalyzed conversion of lignin, and ITC can gain independent information on the extent and rate of lignin conversion. The development of robust assays for characterizing the catalytic activities of laccase on natural lignin substrate is an essential step toward unlocking the potential of lignin biorefinery.
4. Developing a lignin-consolidated process that integrates lignin fractionation using a DES and further lignin valorization in the same DES medium by laccase oxidative degradation is essential. Previously, we had efficiently fractionated lignin using various DESs from several lignocellulosic biomass (Fakayode et al. 2020; Ji et al. 2021; Li et al. 2023; Ma et al. 2022). Recently, a closed-loop biorefinery process by integrating renewable DES with plant genetic engineering (Kim et al. 2019) and a consolidated strategy for lignin degradation employing a bi-enzyme system that consisted of LiPs and AAOs to depolymerize lignin in ILs (Liu et al. 2021a) had been successfully developed. Given two facts, (1) LiPs, AAOs, and laccase are all lignin-degrading enzymes; (2) DES is the 4th generation of ILs, a consolidated process that combines the fractionation and depolymerization of lignin in laccase–DES systems can be foreseen as a highly feasible route. Future endeavors should be devoted to boosting the biocompatibility of DES with laccase to accomplish this prospect. Take ChCl:lactic acid, for example. It is an efficient lignin fractionation media (Zhou et al. 2022a). However, it inhibits *Myceliophthora thermophila* laccase activity (Chan et al. 2021). The inhibitory effect can be alleviated via strategies mentioned in ‘‘Protein engineering to improve the compatibility of DES with laccase’’ Sect, improving the compatibility of ChCl:lactic acid with laccase. Vice versa, the lignin fractionation capability of betaine:lactic acid, which enhances laccase activity, can be improved through solvent engineering methods such as incorporating a ternary component to tailor its solvatochromic properties. Likewise, the biocompatibility of DES with other LDEs should be investigated to explore more enzymatical routes for lignin valorization. Future endeavors should be devoted to boosting lignin enzymatic conversion as more efficient and cost-effective.

## Concluding remarks

Enzymatic transformation of lignin into high-value products holds tremendous potential in the cleaner and more sustainable development of lignin valorization strategies. A growing consensus is that laccase plays a vital role in lignin degradation. Despite this, lignin bioconversion is hindered by its structural heterogeneity and poor water solubility. DES has been proven to be a capable class of lignin solvents and offer greater flexibility for structural manipulation. Recent insights in elucidating the interactions between laccase and DES lay a solid foundation for customizing DESs bearing both efficient lignin dissolution and high biocompatibility. Designing laccase–DES pairs for lignin valorization in the DES medium can be conceivably envisaged and are critical for a current and future sustainable and bio-based economy.

## Data Availability

Not applicable.

## Abbreviations

AAOs: Aryl alcohol oxidases
ABTS: 2,2′-Azino-bis (3-ethylbenzothiazoline-6-sulfonic acid)
CAA: Colorimetric activity assays
CD: Circular dichroism
ChCl: Choline chloride
COSMO-RS: Conductor-like screening model for real solvents
DES: Deep eutectic solvents
DyPs: Dye-decoloring peroxidases
EPR: Electron paramagnetic resonance
GRAS: Generally recognized as safe
HBA: Hydrogen bond acceptor
HBD: Hydrogen bond donor
HBT: 1-Hydroxybenzotriazole
HTS: High-throughput screening
ILs: Ionic liquids
ITC: Isothermal titration calorimetric
LDEs: Lignin degrading enzymes
LiPs: Lignin peroxidases
LMS: Laccase mediator system
MD: Molecular dynamic
MnPs: Manganese peroxidases
NADES: Natural deep eutectic solvents
NIMS: Nanostructure-initiator mass spectrometry
NMR: Nuclear magnetic resonance
OTR: Oxygen transfer rate
SANS: Small-angle neutron scattering
TEMPO: 2,2,6,6-Tetramethyl-1-piperidinyloxy
VPs: Versatile peroxidases
